# 1757. Effectiveness of vaccination against tick-borne encephalitis in the Czech Republic, 2018-2022

**DOI:** 10.1093/ofid/ofad500.1588

**Published:** 2023-11-27

**Authors:** Jan Kyncl, Frederick Angulo, Hana Orlikova, Pingping Zhang, Iva Vlckova, Marek Maly, Dagmar Krivohlavkova, Lisa Harper, Juanita Edwards, Cody Bender, Andreas Pilz, Wilhelm Erber, Harish Madhava, Jennifer Moïsi

**Affiliations:** National Institute of Public Health, Prague, Hlavni mesto Praha, Czech Republic; Pfizer Vaccines, Collegeville, Pennsylvania; National Institute of Public Health, Prague, Hlavni mesto Praha, Czech Republic; Pfizer Vaccines, Collegeville, Pennsylvania; National Institute of Public Health, Prague, Hlavni mesto Praha, Czech Republic; National Institute of Public Health, Prague, Hlavni mesto Praha, Czech Republic; Pfizer Biopharmaceuticals Group, Prague, Hlavni mesto Praha, Czech Republic; Pfizer Vaccines, Collegeville, Pennsylvania; Pfizer Vaccines, Collegeville, Pennsylvania; Pfizer Vaccines, Collegeville, Pennsylvania; Pfizer Corporation Austria, Vienna, Wien, Austria; Pfizer, Vienna, Wien, Austria; Pfizer Vaccines, Collegeville, Pennsylvania; Pfizer Vaccines, Collegeville, Pennsylvania

## Abstract

**Background:**

Tick-borne encephalitis (TBE) is an infection by the tickborne encephalitis virus that causes symptoms of central nervous system inflammation (meningitis, encephalitis, etc.). TBE is endemic in parts of Europe including the Czech Republic. In 2020, 23% of 3,734 TBE cases reported to the European Centre for Disease Prevention and Control were from the Czech Republic. TBE vaccination is universally recommended in the Czech Republic, but TBE vaccine effectiveness (VE) has not been assessed.

**Methods:**

Laboratory-confirmed TBE is a notifiable disease in the Czech Republic with mandatory reporting by clinicians to public health authorities. Vaccine histories of TBE cases were determined by medical record review and interviews. Online household surveys on vaccine uptake were conducted in 2019-2020. A person who received the three-dose primary series and who was not late for the subsequent booster(s) was fully-vaccinated. VE against TBE was determined by comparing the proportion of TBE cases who were fully-vaccinated (PCV) and the proportion of survey respondents who were fully-vaccinated (PPV), excluding partially-vaccinated, using the screening method formula VE=1-[PCV/(1-PCV)]/[PPV/(1-PPV)].

**Results:**

In 2018-2022, 3648 TBE cases were reported in the Czech Republic; 98.1% (3105/3166) of TBE cases with known vaccination history were unvaccinated. The incidence of TBE was 6.8/100,000 population per year (PPY); the highest incidence was in the southwestern Jihočeský region (17.8/100,000 PPY). Of the TBE cases with known hospitalization, 94.5% (3324/3517) were hospitalized. There were 20 (0.55%) deaths. Among 42,671 persons surveyed with known TBE vaccination history, 66.5% were unvaccinated. VE for the prevention of TBE was 97.6% (95% confidence interval 95.7-98.7). By age groups, VE was 97.1% (88.4-99.3) in 1-15 years-of-age, 97.9% (95.3-99.0) in 16-59 years-of-age, and 96.9% (90.5-99.0) in > 60 years-of-age. TBE vaccination averted an estimated 1020 TBE cases in the Czech Republic from 2018-2022.

**Conclusion:**

TBE vaccination was highly effective in preventing TBE in the Czech Republic. The protective effect of TBE vaccines was evident in all age groups. To prevent additional TBE cases in the Czech Republic and elsewhere, efforts to increase TBE vaccine uptake are needed.

**Disclosures:**

**Frederick Angulo, DVM PhD**, Pfizer Vaccines: Employee|Pfizer Vaccines: Stocks/Bonds **Pingping Zhang, MS**, Pfizer Vaccines: Stocks/Bonds **Dagmar Krivohlavkova, M.D.**, Pfizer Vaccine: Employee|Pfizer Vaccine: Stocks/Bonds **Lisa Harper, M.S.**, Pfizer Vaccines: Stocks/Bonds **Juanita Edwards, B.S.**, Pfizer Vaccines: Stocks/Bonds **Cody Bender, M.S.**, Pfizer Vaccines: Stocks/Bonds **Andreas Pilz, PhD**, Pfizer Vaccines: Stocks/Bonds **Wilhelm Erber, PhD**, Pfizer Vaccines: Stocks/Bonds **Harish Madhava, M.D.**, Pfizer Vaccines: Stocks/Bonds **Jennifer Moïsi, PhD**, Pfizer Vaccines: Stocks/Bonds

